# Microbiological Safety Assessment of Fermented Cassava Flour “*Lafun*” Available in Ogun and Oyo States of Nigeria

**DOI:** 10.1155/2013/845324

**Published:** 2013-07-09

**Authors:** A. O. Adebayo-Oyetoro, O. B. Oyewole, A. O. Obadina, M. A. Omemu

**Affiliations:** ^1^Department of Food Technology, Yaba College of Technology, PMB 2011, Yaba, Lagos 101212, Nigeria; ^2^Department of Food Science and Technology, Federal University of Agriculture, PMB 2240, Ogun State, Abeokuta 110001, Nigeria; ^3^Department of Food Service and Tourism, Federal University of Agriculture, PMB 2240, Ogun State, Abeokuta 110001, Nigeria

## Abstract

The microorganisms involved in the fermentation and spoilage of fermented cassava flour were investigated. The water samples used at the different processing sites were also investigated to determine their safety status. There was predominance of *Staphylococcus aureus*, *Aspergillus* spp., and *Escherichia coli* in all samples. Coliforms were observed to be present in all of the processing water. In the fermented cassava flour, the total bacterial count ranged between 4.9 × 10^6^ cfu/mL from Eleso, Bakatari, and Oja Odan processing sites and 8.10 × 10^6^ cfu/mL in Eruku processing site. The majority of the microorganisms involved in the spoilage of “*lafun*” were found to be *Aspergillus niger* which ranged between 4.6 × 10^5^ cfu/mL in Eleso and 8.1 × 10^5^ cfu/mL in Kila. The control sample prepared in the laboratory had a low microbial load compared to samples collected from various sites and markets.

## 1. Introduction

Cassava (*Manihot esculenta C*rantz) is a major root crop in the tropics and its starchy roots are significant sources of calories for more than 500 million people worldwide [1]. It is the most important root crop in Nigeria in terms of food security, employment creation, and income generation for crop-producing households [2]. It supplies about 70% of the daily calories of over 50 million people in Nigeria [3]. 

 Nigeria is the largest producer of cassava in the world [1] with about 45 million metric tonnes and its cassava transformation is the most advanced in Africa [4]. Cassava is grown throughout the tropic and could be regarded as the most important root crop, in terms of area cultivated and total production [5]. It is a major food crop in Nigeria [6]. It is essentially a carbohydrate food with low protein and fat. The edible part of fresh cassava root contains 32–35% carbohydrate, 2-3% protein, 75–80% moisture, 0.1% fat, 1.0% fibre, and 0.70–2.50% ash [3]. The production of cassava for human consumption has been estimated to be 65% of cassava products, while 25% is for industrial use, mostly starch 6% or animal feed 19% and 10% lost as waste [7].

The major uses of cassava in Nigeria include flour, gari, fufu, livestock feeds, confectionaries, monosodium glutamate processing, sweeteners, glues, textiles, and pharmaceuticals. In spite of the desirability of cassava for consumption as food and animal feed, it contains some toxic compounds such as cyanogenic glycosides, linamarin, and lotaustralin which are highly toxic. Thus, the consumption of an inadequately processed cassava product for prolonged periods may result in chronic toxicity. However, the toxicity of the cyanogens is a result of inadequate processing [8]. Cassava roots are highly perishable and a lot of postharvest losses occur to this commodity during storage due to high physiological activities and activities of microorganisms that enter bruises received during harvesting as well as the inherent high moisture content of fresh roots, which promote both microbial deterioration and unfavourable biochemical changes in the commodity [9]. 

**Figure 1:**
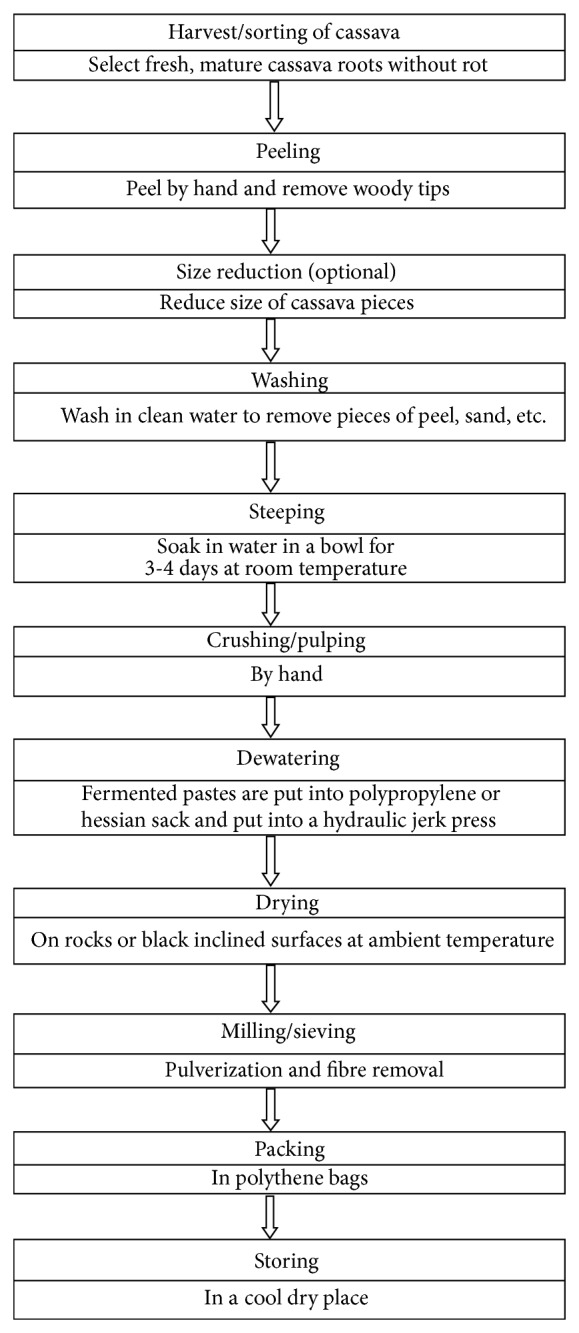
Process diagram for *lafun*.

*Lafun* is a fine flour obtained from the traditional fermentation of cassava (Figure 1). It is usually prepared as a stiff porridge using boiling water, prior to being consumed with soup. The processing involves peeling, cutting, submerged fermentation, dewatering, sun drying, and milling. One of the constraints in the commercialization of locally fermented cassava products is that the quality of the products varies from one processor to the other and even from one processing batch to the other by the processor [10, 11].

Meanwhile, a report on high rate of spoilage as well as toxicity resulting from the consumption of *lafun *has necessitated this research. This study is aimed at evaluating the microbiological safety status of the *lafun* produced in south west Nigeria. 

## 2. Materials and Methods

*Lafun* samples were collected aseptically from the processors and sellers in 10 locations in Ogun and Oyo States. The two states were selected for being the major producers and consumers of this product. The samples after collection were labeled and taken to the laboratory for analysis which is usually not later than 24 hours after collection. Processing water samples from boreholes, rain, and streams were also collected in sterile containers for analysis. 

### 2.1. Microbiological Analysis

The analysis involved total plate count, fungi count, and coliform count. 

### 2.2. Isolation and Identification

Samples of the *lafun *collected were serially diluted tenfold in which ten grams of each sample was diluted in 90 mL peptone water followed by homogenization by horizontal and vertical agitations for a few minutes to obtain 10^−1^ dilution. Further tenfold serial dilutions were made up to 10^−5^ for colony count. 1 mL of volume of each dilution was spread plated in triplicate on de Man Rogosa Sharpe Agar (MRS; Oxoid CM 361) and incubated anaerobically at 35°C for 48 h for the enumeration of lactic acid bacteria, and Plate Count Agar (PCA; Oxoid CM 325) incubated at 32°C for 48 h was used for the enumeration of aerobic bacteria [12]. 0.1 mL of each of the samples was also plated on Potato Dextrose Agar (Oxoid) supplemented with 60 *μ*g mL^−1^ chloramphenicol for fungal isolation. This was incubated at 28°C for 5 days. All plates were prepared in triplicate. The colonies were counted and recorded followed by isolation, purification, and storage on Nutrient Agar (Lab M) slants and kept at 4°C for further characterization and identification [13, 14].

### 2.3. Characterization of Isolates

Bacterial isolates were characterized and identified using series of cultural and biochemical tests such as Gram staining, glucose, indole reaction coagulase, and catalase as described by [15]. 

### 2.4. pH Determination

The pH was determined using Kent pH meter (Kent Ind. Measurement Ltd., UK) model 7020 equipment with a glass electrode as described by [16]. Ten grams of the *lafun* sample was weighed and dissolved in 100 mL sterile distilled water. The solution was later decanted and the pH was measured. The pH of the processing water was also determined using the same equipment. 

### 2.5. Statistical Analysis

The mean of the total viable bacterial count obtained from the fermented cassava flour was subjected to analysis of variance (ANOVA) and the Duncan multiple range test to separate the means. 

## 3. Results and Discussion

Table 1 shows the mean values for the total colony count for bacterial isolates from fermented cassava flour (*lafun*) obtained from the processing sites and markets. Among the organisms isolated are *Escherichia coli, Klebsiella oxytoca, Bacillus cereus, Staphylococcus aureus, and Clostridium sporogenes.*

Table 2 shows the results of the fungal count for the samples obtained from both the processing sites and the markets. It was observed that some of the values were higher than 1.9 × 10^3^ cfu/g to 3.9 × 10^3^ cfu/g as reported by [17] and 0.5–2.5 × 10^3^ cfu/g as reported by [18].

Bacterial isolates from the samples collected were *E. coli, B. cereus, S aureus, K. oxytoca, *and* C. sporogenes* as shown in Table 3. Some of the organisms were Gram positive, catalase positive, and glucose positive. *Bacillus cereus* have been reported to be pathogenic especially if found to be present at 10^5^–10^7^ cfu/g [19]. Meanwhile, sample MNI was found to have the highest amount of aerobic bacterial count of 4.8 × 10^6^ cfu/g, while sample EOS had the highest count of 5.3 × 10^6^ cfu/g among the samples from the markets. These values were higher than the findings of microbial count of the fermented flour obtained by [20] who reported 1.5 × 10^6^ cfu/g. It was also higher than the findings of [17] who reported 2.7 × 10^3^ to 1.2 × 10^7^ cfu/g.

More so, Table 4 shows the aerobic count of stream water used for processing which was found to be the highest in OJO with value of 8.5 ± 1.37 cfu/mL and the lowest in PTJ with 5.5 ± 0.01 cfu/mL. The coliform counts (Table 4) from sites EOS and OJO were found to be the highest with 8.0 ± 1.13 cfu/mL and the lowest in PTJ with 5.3 ± 0.01 cfu/mL. This could be as a result of the same stream water being used by processors for washing and bathing as well as for processing. Some people even defecate in the water thereby reducing the quality of the product. In addition, lactic acid bacteria,* Bacillus *spp. yeast, and filamentous fungi have been shown to be present in traditional fermented cassava (*lafun*) [21]. 

Although water samples used for fermenting previous batches of “*lafun*” have been suggested to be used as back slopping by the processors in the production area [10], this may also account for the high microbial load obtained in these samples. This is due to the possibility of the safety being compromised. The isolation of *Staphylococcus aureus* and *Escherichia coli* from the fermented cassava flour is attributed to postprocessing handling and exposure both at the processing sites and in the markets [22]. This is because the drying is usually done in open air where animals are reared. In some locations, the toilet is constructed near the processing area which could facilitate cross-contamination.

Table 5 shows the results of the pH values for the various samples obtained from the processing sites and markets. The value ranged from 4.05 ± 0.16 in sample EOS to 5.51 ± 0.42 in sample MNI for those samples collected from the processing sites, while the value ranged from 4.08 ± 0.16 in sample EOS to 5.55 ± 0.42 in sample SOP for those collected from the markets. It was observed that the result was comparable to the findings of [23]. Meanwhile, the increase in pH observed in some locations may be due to short period of fermentation by some of these processors thereby limiting the activities of lactic acid bacteria. This is because the product is always in high demand which allows some of these processors to compromise. 

## 4. Conclusion

Our results had shown that although *lafun *is a fermented product, there is possibility of contamination resulting due to poor handling either at the processing site or from the market. In the course of our investigation, we observed that quality is often compromised as some processors make use of back slopping to reduce the length of time for fermentation so as to meet high customers' demand. The displayed pattern in the market also encourages cross-contamination. We suggest provision of pipe borne water or borehole for constant supply of water for processing. We also suggest the provision of modern driers to each community so as to prevent sun drying of *lafun*. 

Finally, it is recommended that close monitoring of the steps involved in processing fermented cassava flour should be carried out by health officials so as to ensure strict compliance by the processors and the sellers in the market.

## Figures and Tables

**Table 1:** Mean values for the total aerobic colony count for bacterial isolates.

Sampling points/code	Total colony count for aerobic bacteria (cfu/g) ×10^6^ (processing sites)	Total colony count for aerobic bacteria (cfu/g) ×10^6^ (markets)
ERK	4.0 ± 0.07	4.0 ± 0.07
OJO	3.0 ± 0.02	4.0 ± 0.07
OWO	4.3 ± 0.23	4.8 ± 0.73
BTS	3.1 ± 0.09	3.2 ± 0.08
EOS	4.5 ± 0.43	5.3 ± 1.23
SOP	4.2 ± 0.13	4.8 ± 0.73
MNI	4.8 ± 0.73	4.8 ± 0.73
TVE	4.5 ± 0.43	4.7 ± 0.63
PTJ	4.2 ± 0.13	4.8 ± 0.73
OSJ	4.1 ± 0.03	4.5 ± 0.43
CTR	1.1 ± 0.11	

Results are presented as mean values of triplicate samples.

**Table 2:** Mean values for the total colony count for fungal isolates.

Sampling points/code	Total colony count for fungi (cfu/g) ×10^3^ (processing sites)	Total colony count for fungi (cfu/g) ×10^3^ (markets)
ERK	2.8 ± 0.27	3.4 ± 0.34
OJO	3.0 ± 0.29	3.3 ± 0.33
OWO	2.3 ± 0.22	2.2 ± 0.22
BTS	3.4 ± 0.34	4.0 ± 0.39
EOS	2.2 ± 0.22	3.3 ± 0.32
SOP	3.7 ± 0.37	4.0 ± 0.39
MNI	4.0 ± 0.39	3.9 ± 0.39
TVE	3.9 ± 0.40	4.0 ± 0.39
PTJ	3.9 ± 0.38	4.0 ± 0.39
OSJ	3.5 ± 0.30	3.4 ± 0.34
CTR	NIL	

Results are presented as mean values of triplicate samples.

**Table 3:** Biochemical characteristics of bacterial isolates.

Bacteria found	Gram staining	Glucose	Indole	Coagulase	Catalase
*Escherichia coli *	−	+	+	NA	+
*Klebsiella oxytoca *	−	+	+	NA	+
*Bacillus cereus *	+	+	+	NA	+
*Staphylococcus aureus *	+	+	−	+	+
*Clostridium sporogenes *	+	+	−	NA	−

**Table 4:** Microbiological status of water used at the processing sites.

Location	Aerobic cfu/mL	Coliform cfu/mL
ERK	7.3 ± 0.17	7.0 ± 0.2
OJO	8.5 ± 1.37	8.0 ± 1.13
OWO	7.7 ± 0.57	7.2 ± 0.33
BTS	6.4 ± 0.07	6.1 ± 0.08
EOS	8.3 ± 1.17	8.0 ± 1.13
SOP	8.1 ± 0.97	7.9 ± 1.03
MNI	7.5 ± 0.37	7.1 ± 0.23
TVE	6.0 ± 0.01	5.8 ± 0.01
PTJ	5.5 ± 0.01	5.3 ± 0.01
OSJ	6.0 ± 0.11	6.3 ± 0.57
CTR	1.2 ± 0.1	1.1 ± 0.1

**Table 5:** Results of the pH values for *lafun* obtained from processing sites and markets.

Sample codes	Processing site	Market
ERK	4.51 ± 0.34	4.54 ± 0.33
OJO	4.42 ± 0.32	4.42 ± 0.32
OWO	4.24 ± 0.24	4.20 ± 0.22
BTS	4.15 ± 0.20	4.16 ± 0.21
EOS	4.05 ± 0.16	4.08 ± 0.16
SOP	5.50 ± 0.45	5.55 ± 0.42
MNI	5.51 ± 0.42	5.50 ± 0.40
TVE	5.23 ± 0.40	5.52 ± 0.41
PTJ	4.63 ± 0.36	5.53 ± 0.42
OSJ	4.55 ± 0.34	5.51 ± 0.42

## References

[1] FAO Corporate Document Repository. http://www.fao.org/docrep/005/Y4636E/y4636e05.htm.

[2] Ugwu B. O., Ukpabi U. J. (2002). Potential of soy-cassava flour processing to sustain increasing cassava production in Nigeria. *Outlook on Agriculture*.

[3] Oluwole O. B., Olatunji O. O., Odunfa S. A. (2004). A process technology for conversion of dried cassava chips into gari. *Journal of Food Science and Technology*.

[4] Egesi C., Mbanaso E., Ogbe F., Okogbenin E., Fregene M. (2006). Development of cassava varieties with high value root quality through induced mutations and marker-aided breeding. *NRCRI, Umudike Annual Report*.

[5] Ano A. O. (2003). Studies on the effect of Liming on the Yield of two cassava cultivars. *NRCRI Annual Report*.

[6] Ogbe F. O., Emehute J. K. U., Legg J. (2007). Screening of cassava varieties for whitefly populations. *NRCRI Annual Report*.

[7] Fish D. M., Trim D. S. (1993). A review of research in the drying of cassava. *Tropical Science*.

[8] Bradbury J. H., Cumbana A., Mirione E., Cliff J. (2006). Reduction of cyanide content of cassava flour in Mozambique by the wetting method. *Food Chemistry*.

[9] Wenham J. E. (1995). Post-harvest deterioration of cassava. A Biotechnology perspective. *F.A.O. Plant Production and Protection Paper*.

[10] Padonou S. W., Hounhouigan J. D., Nago M. C. (2009). Physical, chemical and microbiological characteristics of *lafun* produced in Benin. *African Journal of Biotechnology*.

[11] Oyewole O. B., Sanni L. O., Agbor Egbe T., Bauman A., Griffon T., Treche S. (1995). Constraints in traditional cassava food processing: the case of fufu production. *Cassava Food Processing*.

[12] Owusu-Kwarteng J., Tano-Debra K., Glover R. L. K., Akabanda F. (2010). Process characteristics and Microbiology of *fura* produced in Ghana. *Nature and Science*.

[13] Somorin Y. M., Bankole M. O., Omemu A. M., Atanda O. O. (2011). Impact of milling on the microbiological quality of yam flour iii southwestern Nigeria. *Research Journal of Microbiology*.

[14] Obadina A. O., Oyewole O. B., Odusami A. O. (2009). Microbiological safety and quality assessment of some fermented cassava products (*lafun*, *fufu* , *gari*). *Scientific Research and Essays*.

[15] Ochei J., Kolhatkar A. (2008). *Medical Laboratory Science: Theory and Practice*.

[16] Oyewole O. B., Odunfa S. A. (1990). Characterization and distribution of lactic acid bacteria in cassava fermentation during fufu production. *Journal of Applied Bacteriology*.

[17] Tsav-Wua J. A., Inyang C. U., Akpapunam M. A. (2004). Microbiological quality of fermented cassava flour ‘kpor umilin’. *International Journal of Food Sciences and Nutrition*.

[18] Eleazu C. O., Amajor J. U., Ikpeama A. I., Awa E. (2011). Studies on the nutrient composition, antioxidant activities, functional properties and microbial load of the flours of 10 Elite cassava (*Manihot esculenta*) varieties. *Asian Journal of Clinical Nutrition*.

[19] Michelet N., Granea P. E., Mahillon J., Alouf J. E., Popoff M. R. (2006). *Bacillus cereus* enterotoxins, bi- and tri-component cytolysins, and other hemolysins. *The Comparative Sourcebook of Bacterial Protein Toxins.*.

[20] Ijabadeniyi A. O. (2007). Microbiological safety of gari, *lafun* and ogiri in Akure metropolis, Nigeria. *African Journal of Biotechnology*.

[21] Oyewole O. B., Ayo Odunfa S. (1989). Effects of fermentation on the carbohydrate, mineral, and protein contents of cassava during “fufu” production. *Journal of Food Composition and Analysis*.

[22] Obadina A. O., Oyewole O. B., Sanni L. O., Tomlins K. I., Westby A. (2008). Identification of hazards and critical control points (CCP) for cassava fufu processing in South-West Nigeria. *Food Control*.

[23] Hongbete F., Mestres C., Akissoe N., Nago M. C. (2009). Effect of processing conditions on cyanide content and colour of cassava flours from West Africa. *African Journal of Food Science*.

